# Can money heal all wounds? Social exchange norm modulates the preference for monetary versus social compensation

**DOI:** 10.3389/fpsyg.2015.01411

**Published:** 2015-09-23

**Authors:** Yulong Cao, Hongbo Yu, Yanhong Wu, Xiaolin Zhou

**Affiliations:** ^1^Center for Brain and Cognitive Sciences and Department of Psychology, Peking UniversityBeijing, China; ^2^Key Laboratory of Machine Perception (Ministry of Education), Peking UniversityBeijing, China; ^3^Beijing Key Laboratory of Behavior and Mental Health, Peking UniversityBeijing, China; ^4^PKU-IDG/McGovern Institute for Brain Research, Peking UniversityBeijing, China

**Keywords:** social exchange norm, interpersonal transgression, compensation, individual differences

## Abstract

Compensation is a kind of pro-social behavior that can restore a social relationship jeopardized by interpersonal transgression. The effectiveness of a certain compensation strategy (e.g., repaying money, sharing loss, etc.) may vary as a function of the social norm/relationship. Previous studies have shown that two types of norms (or relationships), monetary/exchange and social/communal, differentially characterize people’s appraisal of and response to social exchanges. In this study, we investigated how individual differences in preference for these norms affect individuals’ perception of others’ as well as the selection of their own reciprocal behaviors. In a two-phase experiment with interpersonal transgression, we asked the participant to perform a dot-estimation task with two partners who occasionally and unintentionally inflicted noise stimulation upon the participant (first phase). As compensation one partner gave money to the participant 80% of the time (the monetary partner) and the other bore the noise for the participant 80% of the time (the social partner). Results showed that the individuals’ preference for compensation (repaying money versus bearing noise) affected their relationship (exchange versus communal) with the partners adopting different compensation strategies: participants tended to form communal relationships and felt closer to the partner whose compensation strategy matched their own preference. The participants could be differentiated into a social group, who tended to form communal relationship with the social partner, and a monetary group, who tended to form communal relationship with the monetary partner. In the second phase of the experiment, when the participants became transgressors and were asked to compensate for their transgression with money, the social group offered more compensation to the social partners than to the monetary partners, while the monetary group compensated less than the social group in general and showed no difference in their offers to the monetary and social partners. These findings demonstrate that the effectiveness of compensation varies as a function of individuals’ preference for communal versus monetary norm and that monetary compensation alone does not heal all wounds.

## Introduction

What would you do if you forgot your mother’s birthday? You may feel guilty and make up for this by spending more time with her (de Hooge et al., 2011). Compensation following social/interpersonal transgression helps to restore the threatened social relationship and reinstate social justice (Baumeister et al., 1994). There are many forms of compensation, such as monetary compensation and liability sharing. Different types of compensation are not equally effective in restoring the jeopardized relationship in all social contexts. For example, your disappointed mother in the above example will not be happier if you pay her for forgetting her birthday. In a similar vein, if you break a vase in a souvenir shop, it is better to pay rather than just make an apology (at least in some cultures). What are the factors that influence the effectiveness of compensation? This question is of great societal, political, and philosophical significance (Lazare, 2004), as it is crucial to important issues such as a victim’s reception of institutional or personal compensation. For example, what is the optimal way to compensate the survivors of holocaust or the victims of racial discrimination? Can money heal all wounds? If not, what might be the factors that influence the effectiveness of monetary/material compensation?

Obviously, the offering and reception of compensation involve a set of socioeconomic and psychological processes governed by certain social norms or social relationships. In social psychology, an influential theory that characterizes different norms/relationships is the monetary/exchange and social/communal dichotomy proposed by Heyman and Ariely (2004). They argued that the monetary norm prompts people to be highly sensitive to the magnitude of the compensation, balancing the debt precisely and immediately, usually in calculable ways; in contrast, the social or communal norm does not demand reciprocity with such urgency and precision, but focuses more on the “soft” aspects of social interaction, such as mutual understanding, mutual support, and long-term relationship. As for the psychological mechanism through which the exchange/communal rules influences people’s social behavior, the self-signaling theory posits that an action is chosen in part to secure good news about the inner traits or abilities one values (Bodner and Prelec, 2003). Viewed in this way, one may choose to reciprocate a social encounter so that such reaction could reveal his/her committed social norms (e.g., exchange versus communal).

The monetary/exchange versus social/communal dichotomy captures a stable difference in individuals’ appraisal and behavior in social interactions. Is it possible then that individuals’ preference of monetary versus communal relationship influences how they perceive a specific form of compensation and how they make compensations to others in interpersonal transgression? Few study have focused on such individual differences. Nevertheless, a hint for the answer to this question comes from research on gift-giving, which has shown how giver’s and recipient’s characteristics influence the effectiveness of gift-giving. For instance, some gift-giving researchers explored the effect of the match between the gift and the recipient’s desire or taste related to gift appreciation (Gino and Flynn, 2011; Ward and Broniarczyk, 2011); other researchers explored the effect of the relationship between the giver and the recipient and its effects on gift appreciation (Belk, 1976; Ruth et al., 1999). Recently a number of researchers also focused on how the degree of match between a gift and the giver’s identity influences recipients’ appreciation (Paolacci et al., 2015). To a certain extent compensation can be viewed as a kind of ‘giving’ in the context of interpersonal transgression. We thus hypothesize that individuals’ preference differences for social versus monetary norms may modulate their attitude toward the compensation given to them as well as their own choice of compensation when they harm others.

In this study, we tested this hypothesis by carrying out an experiment in which participants interacted with two partners (confederates) in a two-phase game with interpersonal transgression and compensation. The research question we are specifically interested in is whether and how preference in communal versus monetary ways of social interaction influences individuals’ social relationships and reciprocal behaviors. In the first phase, the participant interacted with each of the partners in consecutive blocks. Each of these blocks consisted of several rounds of a game in which the partner estimated the number of dots rapidly presented on the screen. If the partner’s estimation was correct, no punishment was delivered and the next round began. If the partner’s estimation was incorrect, the participant had to bear a noise stimulus that was moderately unpleasant (for details of this task, see Yu et al., 2014). In the rounds in which the participant had to receive the noise punishment, the partner could choose to compensate the participant either by allocating an amount of money to the participant or by bearing the noise stimulus for the participant. In fact, the partners’ choice was predetermined by the experimenters such that one partner (the ‘social partner’) chose to bear the noise for the participant 80% of the time and the other partner (the ‘monetary partner’) chose to allocate money 80% of the time. In the second phase, the roles of the participant and the partners were reversed and the participant could only compensate his/her partner by allocating money. If the participant’s dot estimation was incorrect, the partner received pain stimulation. In that case, the participant could subsequently choose to allocate an amount of money from his/her pie to the partner as compensation. We predict that individuals who prefer their social interactions to be governed by exchange norm may form closer relationship with and exhibit more reciprocal behaviors toward the partner who interacts with them under exchange norm; similarly, individuals who prefer communal norm may feel closer to and exhibit more reciprocal behaviors toward partners who also behave in accordance with the communal norms.

## Materials and Methods

### Participants

Seventy-seven participants participated in the experiment. Three were excluded for failing the manipulation check, leaving 74 participants (37 females; age range: 18–26, mean age = 22.6, *SD* = 1.8) in the data analysis. We only recruited participants who had never taken part in experiments involving social interactions with other players. None of the participants reported any history of chronic pain or mental disorders. They all gave informed consent prior to the experiment. This study was conducted in accordance with the Declaration of Helsinki and was approved by the Ethics Committee of the Department of Psychology, Peking University.

### Experimental Design and Procedure

Each participant interacted with two partners (confederates) in a two-phase interpersonal transgression game. Upon arrival the participant first met the two confederates (partners; one male, one female) and was told that they would play an interactive game together through the intranet but in separate rooms. To increase the verisimilitude of the interaction context, the participant and two confederates drew a lottery to decide who underwent the dot-estimation task first. The two confederates were always assigned the role to do the estimation task first and were subsequently guided to another testing room, leaving the participant alone in the room with the experimenter. The participant was told that all the three players were endowed with 500 tokens each. This amount could increase or decrease during the interactive games, and the final tokens one held at the end of the game would be exchanged for a monetary bonus after the experiment (100 tokens amounted to ¥1, ∼ $ 0.2). The participant was told in advance that there would be a second phase of the game and the roles of the participant and the partners would be reversed in the second phase.

The participant then underwent calibrations of noise-bearing and pain stimulation. The participant received noise stimulation as a consequence of the partner’s erroneous response in the first phase. The intensity of the stimulus was calibrated individually so that it was unpleasant but bearable. Specifically, after the participant put on the earphones, we gradually increased the intensity of the noise stimuli until the participant reported ‘moderately unpleasant’ noise levels. This intensity was used in the first phase of the experiment. To keep consistent with the paradigm of measuring the compensatory behavior from a previous study (Yu et al., 2014), the partner received electrical stimulation as the negative result of the participant’s erroneous response in the second phase of the experiment. Given this, we asked the participant to experience the pain stimulation so that he/she would be more likely to trust our manipulation and clearly experience different pain levels. An intra-epidermal needle electrode was attached to the left wrist of the participant for cutaneous electrical stimulation (Inui et al., 2002). Participant-specific pain threshold was calibrated and three levels of pain stimulation were set, corresponding to 0, 4, and 8 on a 0 – 10 scale (0: ‘no sensation at all’; 10: ‘unbearable pain’). We used aversive physical stimulation (noise and pain) rather than monetary loss as interpersonal harm for a number of reasons. First, we aimed to compare the effectiveness of different compensation strategies on interpersonal relationship and reciprocal behavior. If the interpersonal harm is monetary, it does not make much sense to compensate in a non-monetary manner. Second, when measuring the participants’ reciprocal/compensatory behavior, it is important to make the harm and compensation orthogonal, otherwise the salient fairness norm could strongly bias the behavioral measure.

#### The First Phase

**FIGURE 1:**
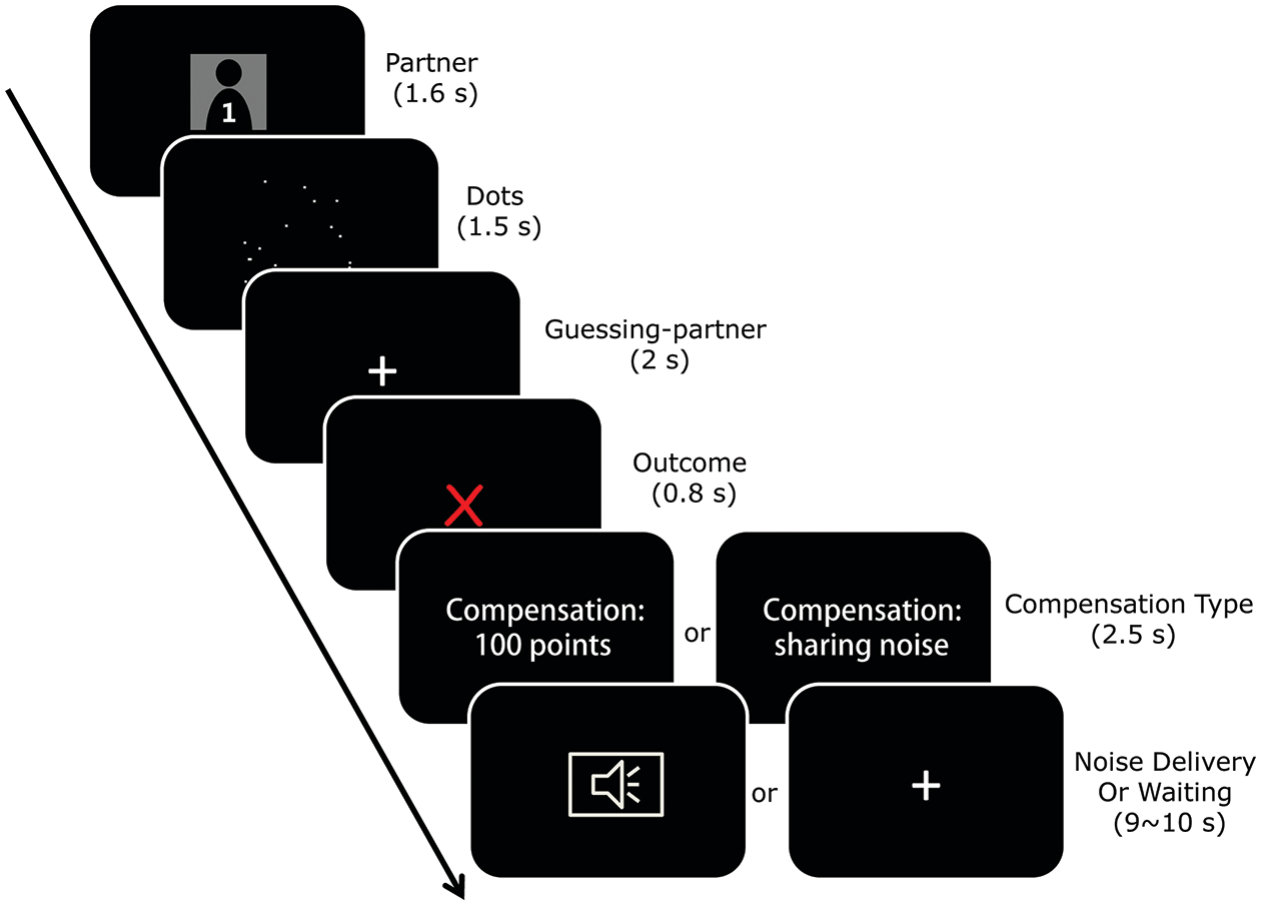
**The task in the first phase.** Each trial began with a fixation and then a cue indicating which partner was paired with the participant for the current trial. The participant was told that his/her partner had to quickly estimate the number of dots on the screen by pressing a corresponding button to indicate whether his/her estimation was more or less than a number (randomly chosen from 19, 20, and 21) which appeared on the next screen. The outcome of the estimation (correct versus incorrect) was communicated to the participant on the next screen. After a correct performance, the partner received 100 monetary tokens as a reward and the next round began. After an incorrect performance, the participant was threatened with the possibility of receiving noise stimulation, and the partner had the chance to choose from two compensation options: paying 100 tokens to the participant or bearing the noise for the participant. The partner’s decision was communicated to the participant on the screen. Finally, the noise stimulation was delivered to the participant if his/her partner decided to pay money, or to his/her partner if the partner decided to bear the noise stimulation for the participant.

In the first phase of the study (**Figure 1**), the participant was told to pay attention to their partners’ behavioral pattern. The partner’s identity was indicated by a number (i.e., Partner 1 or Partner 2), thus preventing the participant from knowing the partner’s true identity. In each round of the game, one partner was randomly chosen to interact with the participant. The participant was told that this partner underwent a dot-estimation task (Yu et al., 2014): if the partner estimated incorrectly, the participant was administered a moderate but unpleasant noise stimulation for 10 s. The noise stimulation was induced by a pair of earphones linked to a computer at a fixed volume calibrated before the experiment. Before the noise delivery, the partner could choose to compensate the participant by either allocating 100 tokens to the participant or by bearing the noise for the participant. The participant would avoid the noise but not receive the 100 tokens if the partner chose to bear the noise, or they would receive 100 tokens but bear the unpleasant noise if the partner chose to pay money. Note that the feedback of the performance on the dot-estimation task and partners’ choice of compensation were predetermined by a computer program so that the partners’ accounts were always enough to pay the 100 tokens as compensation during the game. If the partner estimated correctly, the partner received 100 tokens as a reward. Specifically, the partner’s choice of compensation was predetermined so that Partner 1 chose monetary compensation 80% of the time (monetary condition) and Partner 2 chose noise-bearing 80% of the time (social condition). Hereafter, we refer to Partner 1 as the ‘monetary partner’ and Partner 2 as the ‘social partner’. The word ‘social’ is employed only to signify the compensation type (i.e., bearing noise). The first phase of the study consisted of 60 trials (30 for each partner) and lasted for about 15 min. Specifically, Partner 1 (the ‘monetary partner’) estimated correctly in 15 trials (fillers) and incorrectly in the other 15 trials. In the latter trials, the monetary partner chose monetary compensation in 12 trials and noise-bearing in three trials. Similarly, the social partner estimated correctly in 15 trials and incorrectly in 15 trials. In contrast to the monetary partner, the social partner chose noise-bearing in 12 trials and monetary compensation in three trials. Note, given that the noise stimulation was presented to the participant only sporadically, adaptation to noise was minimal and the adverse effect of the noise stimulation was maintained over the first phase.

After the first phase, the participant answered questions about the two partners’ compensation behavior. The participant was asked whether each of the partners had a preference for the type of compensation and what was that preference. Three participants were discarded because they did not answer these questions correctly. The participant’s general preference for the two types of compensation (paying money versus bearing noise) was measured with two questions (“to what extent you prefer your partner compensates you with money” and “to what extent you prefer your partner bear the noise for you”). The difference between the preference scores (paying money minus bearing noise) was used in the following analysis. The participant’s perceived closeness (or social distance) with respect to each of the two partners was measured with two questions (“to what extent do you prefer your partner to be your roommate” and “to what extent do you prefer your partner to be your friend”) adapted from Bogardus (1933)^1^. The participant answered these questions on 9-point Likert scales (1 = extremely uncharacteristic, 9 = extremely characteristic). Finally, the participant’s willingness to form an exchange relationship with each of the two partners was measured using a questionnaire adapted from Clark and Mills (2011). Four items that were best squared with the current interactive context were chosen from the original 9-item scale (**Table 1**; scale anchor is the same as the closeness rating). High scores on this scale indicate a high exchange (i.e., low communal) relationship. The questions concerning closeness and exchange relationship were given to the participant twice: once for the social partner and once for the monetary partner. Thus, unlike the ordinary personality questionnaires that measure stable and general response tendencies, here the participant had to consider the appropriateness of the statements in relation to the two specific partners. This may have introduced additional inter-item variance into the ratings. Since we were mainly interested in the differential scores between the social and monetary partners, we calculated the reliability based on the differential scores. For the two questions concerning interpersonal closeness, the Cronbach’s α = 0.96. For the four questions concerning exchange relationship, the Cronbach’s α = 0.68. Although the reliability for the exchange relationship questions was not very high^2^, these questions did capture important intra- (social versus monetary partner) and inter-participant (social versus monetary participant subgroup) variability in the exchange/communal relationship (see Results). We admit that these survey questions could potentially make the participants’ attitudes and behavioral tendency toward different partners more consciously available; however, it is unlikely that these questions could reverse such attitudes and tendencies. Moreover, due to the specific question we were interested in (i.e., how the preference of communal versus exchange way of social interaction influences social relationship and reciprocal behavior), we were not able to balance the sequence of our different tasks and surveys.

**Table 1:** Exchange Relationship Scale.

Items
(1) When I give something to another person, I generally expect something in return.
(2) I don’t bother to keep track of benefits that I have given to others.
(3) It is best to make sure things are always kept ‘even’ between two people in a relationship
(4) When I receive benefits from partner, I ought to repay right away.

*Participants rated each item on a 9-point scale (1: extremely uncharacteristic; 9: extremely characteristic) to describe their relationship with each partner.*

#### The Second Phase

**FIGURE 2:**
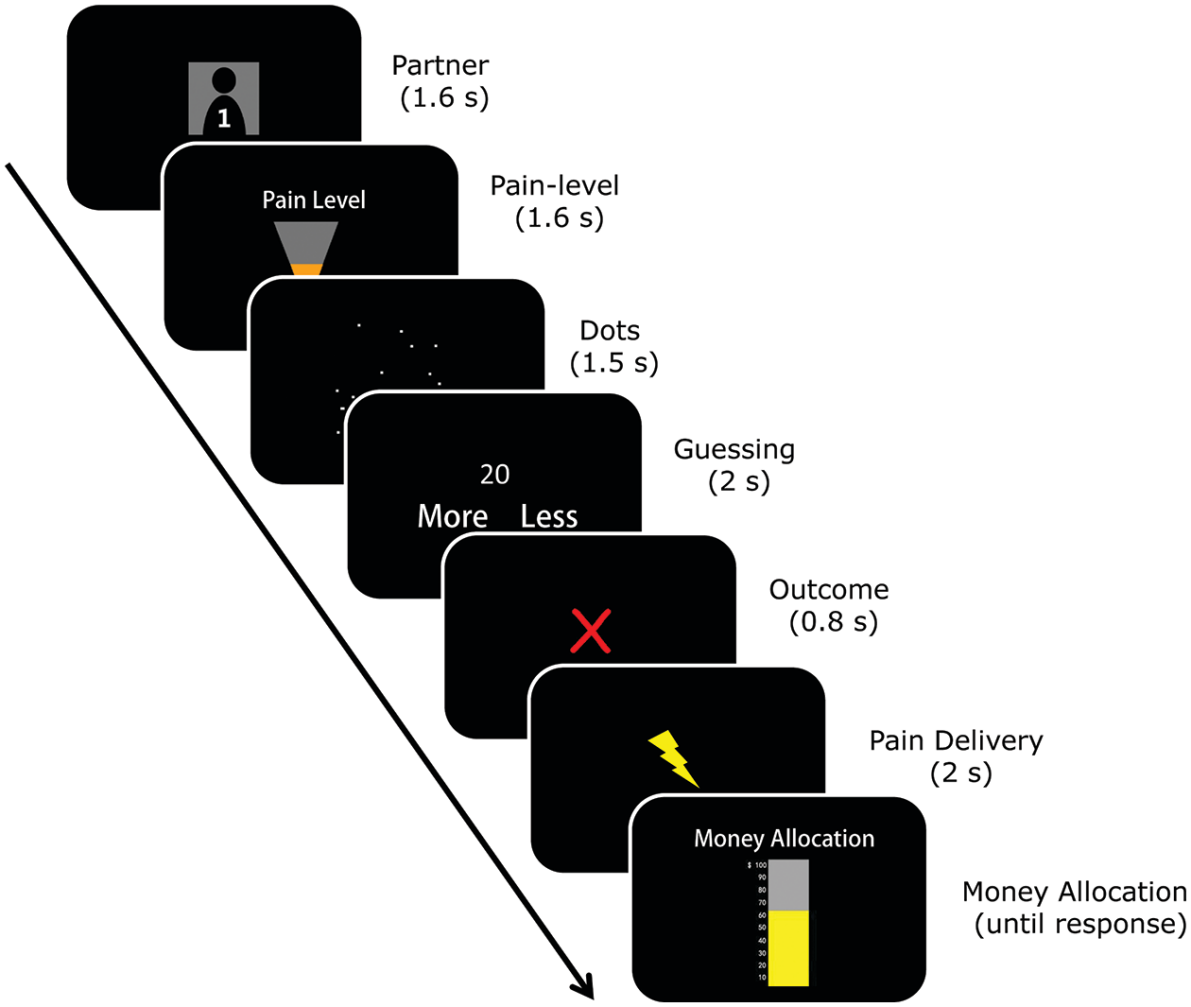
**The task in the second phase.** Each trial began with a cue indicating which of the two partners had been chosen for that particular round. The next screen presented the pain-level of the current trial (none/low/high). Then the participant performed the dot-estimation task. The outcome of the performance was communicated to both the participant and the partner on the next screen. After a correct performance, the participant received 100 monetary tokens as a reward, and the next round began. After an incorrect performance, the partner had to bear pain stimulation. Finally, the participant indicated the amount of monetary tokens (out of 100) he/she would be willing to pay out of his/her own pocket to compensate the partner.

In the second phase of the study (**Figure 2**), the roles of the participant and the partners were reversed; the participant was informed that the partners were not aware of the role-change until then. The participant was then told that in each round, his/her partner had to bear a pain stimulation if he/she (i.e., the participant) estimated incorrectly. The intensity of the electrical stimulation for the partner was randomly chosen from three levels (none/low/high) for each round of the game. The level of pain stimulation delivered to the partner in that trial was communicated to the participant. After pain delivery, the participant decided how many monetary tokens (between 0 and 100) he/she would like to transfer to the partner as compensation. Note, the participant could compensate the partner only by allocating money. The participant was also told that he/she would get 100 tokens as a reward (and the partner would not receive pain stimulation) if he/she made a correct estimation. Thus participant’s account was always sufficient to pay 100 tokens in each round. Unbeknownst to the participant, the feedback of the performance was predetermined. Specifically, there were 72 trials (36 for each partner) in the second phase of study. For the interaction with each partner, there were 18 rounds in which the participant responded correctly (fillers) and 18 rounds in which the participant responded incorrectly. For the latter rounds, there were six rounds in which the partners had to receive high pain stimulation, six rounds of low pain stimulation, and another six rounds of no pain stimulation. On average, the participant could make ¥45 (∼ $ 8; ¥40 for show-up and about ¥5 for bonus).

Before the participant left the lab, he/she answered a set of open questions such as “What do you think about your partners?” and “Do you have any suggestions for improving the interactive settings?” This was to make sure that the participant was not suspicious of our experimental setup. No participant expressed suspicion of the experimental setup or interactive nature of the game.

## Results

### The First Phase

**FIGURE 3:**
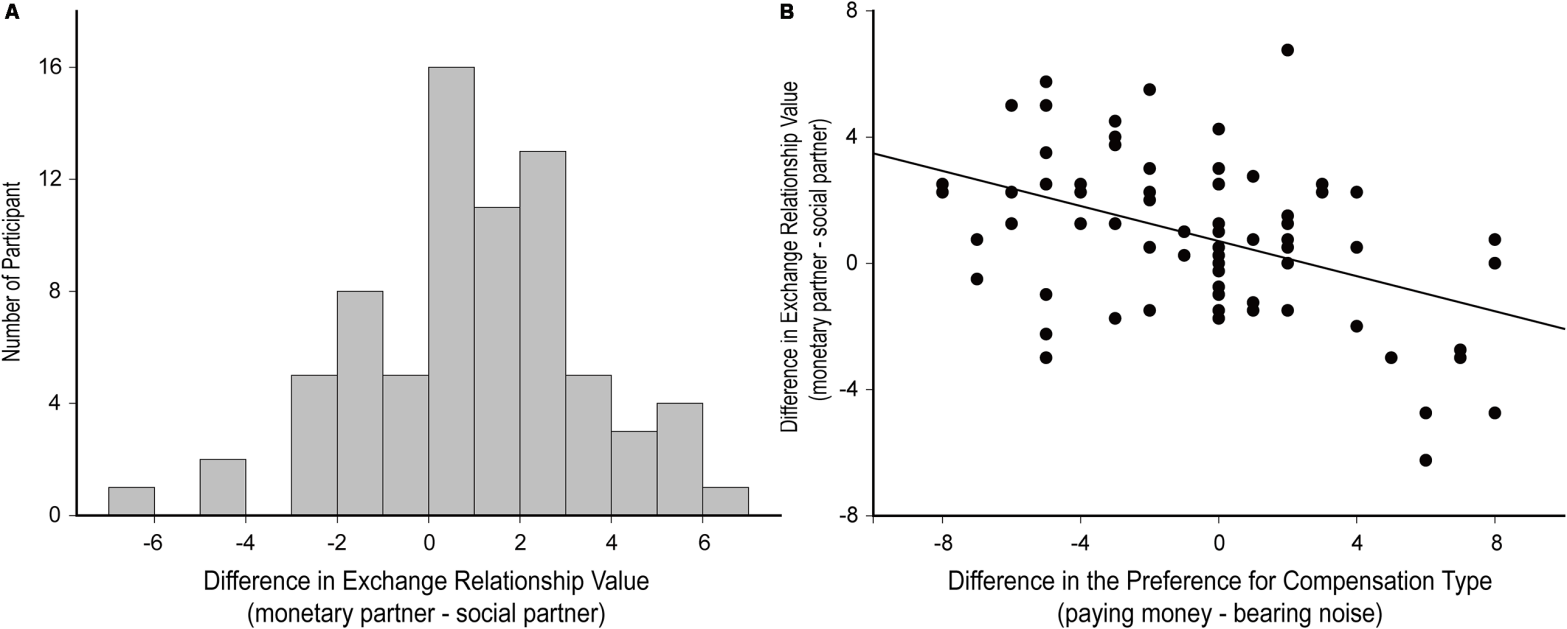
**Individual differences in the preference of compensation and social relationship (exchange versus communal). (A)** The frequency distribution of the difference in participants’ exchange relationship value toward the monetary partner versus social partner. **(B)** The correlation between the difference in exchange relationship value (the monetary partner minus social partner) and the difference in preference for the two compensation types (paying money versus bearing noise).

In the first phase of the study, the participants showed large variability regarding with whom they preferred to form the social versus exchange relationship. To quantify this variability, we computed a score for exchange relationship preference by subtracting the perceived exchange relationship value for the social partner from that for the monetary partner. **Figure 3A** illustrates the distribution of this score over participants. The individual differences most likely resulted from the participants’ preference for compensation type, as indicated by a significant correlation between the difference of participants’ relative preference for each compensation type in general (paying money minus bearing noise) and their difference in exchange relationship value toward the monetary and social partners, *r* = -0.45, *p* < 0.001 (**Figure 3B**).

Thus before we carried out further analyses, we first categorized the participants into two subgroups by median-splitting the participants according to the exchange relationship preference score. This resulted in a low-score subgroup (i.e., the monetary group; *n* = 37) who had a high exchange relationship with the partner bearing the noise and a low exchange relationship with the partner compensating money, and a high-score subgroup (i.e., the social group; *n* = 37) who had a high exchange relationship with the partner compensating money and a low exchange relationship with the partner bearing the noise.

**FIGURE 4:**
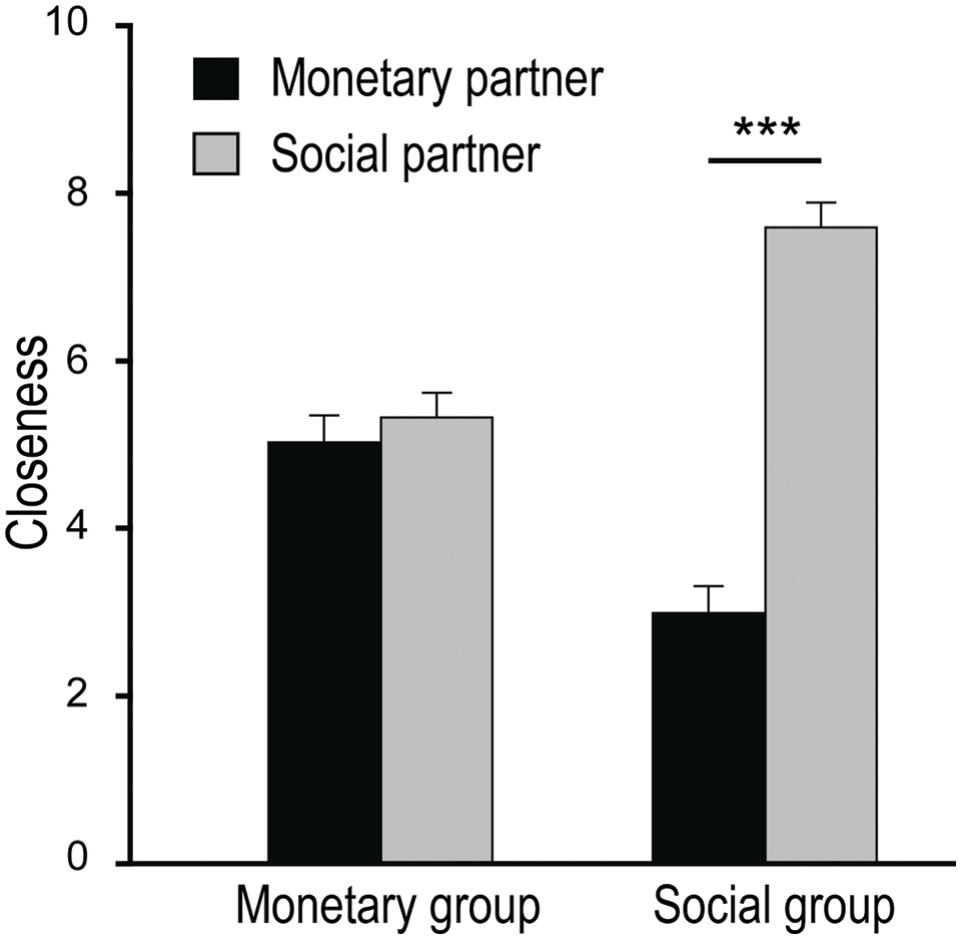
**The closeness ratings as a function of the participant subgroup (monetary versus social) and the partner compensation type (monetary versus social).** Larger score means closer interpersonal relationship. Error bars indicate standard errors. The asterisks denote the significance level of the simple effect ^∗∗∗^*p* < 0.001.

Then we set out to test whether the individual differences in exchange relationship value influenced the participants’ perceived social distance (or, conversely, closeness) with each partner. We carried out repeated measures ANOVA with subgroup (monetary versus social) as a between-subject factor and the partner’s compensation type (paying money versus bearing noise) as a within-subject factor. The main effect of partner’s compensation type was significant, *F*(1,72) = 36.89, *p* < 0.001, $\eta_{p}^{2}$ = 0.34. That is, in general, the participants felt closer with the partner who compensated by bearing the noise (6.5 ± 0.2) than with the partner who compensated by paying money (4.0 ± 0.2). More importantly, the interaction between participant sub-group and partner’s compensation type was significant, *F*(1,72) = 28.49, *p* < 0.001, $\eta_{p}^{2}$ = 0.28 (**Figure 4**). Specifically, for the monetary group, the partner’s compensation types did not influence feelings of closeness, *t*(36) < 1, *p* > 0.1; but for the social group, the closeness with respect to the social partner was significantly higher than that with the monetary partner, *t*(36) = 12.60, *p* < 0.001.

### The Second Phase

**FIGURE 5:**
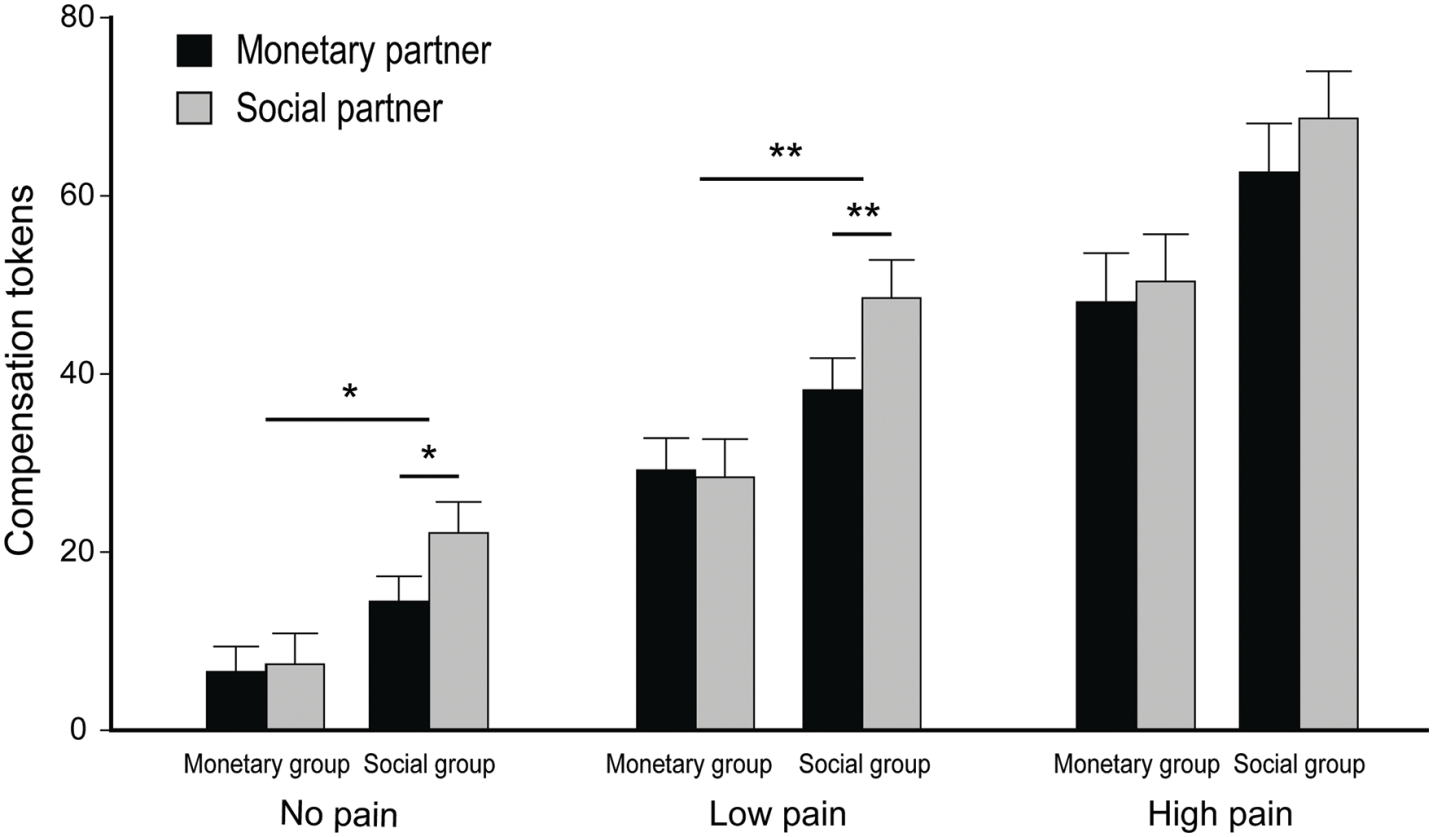
**The amount of monetary compensation to the two partners as a function of the participant subgroup (monetary versus social), the partners’ compensation strategy (paying money versus bearing pain) and pain-level (no/low/high).** Error bars indicate standard errors. The upper asterisks denote the significance level of the two-way interactions, while the lower asterisks denote the significance level of the simple effects. ^∗^*p* < 0.05, ^∗∗^*p* < 0.01.

The second phase of the study provided us with the opportunity to examine how participants’ own compensation behavior could be modulated by the relationship formed with each partner. To this end, we performed a repeated measures ANOVA on the monetary tokens that the participants allocated to the partner as compensation, with participant sub-group (monetary versus social) as a between-subject factor, and the partner’s compensation type in the first phase (paying money versus bearing noise) and pain-level (none/low/high) as within-subject factors. The three-way interaction was marginally significant, *F*(2,144) = 2.95, *p* = 0.056, $\eta_{p}^{2}$ = 0.04 (**Figure 5**). Specially, in the no pain condition the interaction between participant subgroup and the partner’s compensation type was significant, *F*(1,72) = 5.47, *p* < 0.05, $\eta_{p}^{2}$ = 0.07. For the monetary group, the amount of compensation did not differ between the two partners (6.6 ± 2.8 for the monetary partner, 7.4 ± 3.5 for the social partner), *t*(36) = 1.14, *p* > 0.1; for the social group, more compensation was offered to the social partner (22.1 ± 3.5) than to the monetary partner (14.4 ± 2.8), *t*(36) *=* 2.70, *p* < 0.05. At the low pain-level, the interaction between participant group and the partner’s compensation type was also significant, *F*(1, 72) = 10.43, *p* < 0.01, $\eta_{p}^{2}$ = 0.13. For the monetary group, the amount of compensation did not differ between the two partners (29.2 ± 3.6 for the monetary partner, 28.4 ± 4.3 for the social partner), *t*(36) < 1, *p* > 0.1; for the social group, more compensation was offered to the social partner (48.5 ± 4.3) than to the monetary partner (38.2 ± 3.6), *t*(36) = 3.49, *p* < 0.01. In the high pain condition, the interaction between participant group and the partner’s compensation type was not significant, *F*(1,72) < 1, *p* > 0.1. As can be seen from **Figure 5**, this three-way interaction was primarily driven by the lack of differential compensation toward the monetary and the social partner by the social group in the high pain condition. In fact, these participants made very high compensation (about 70 tokens out of 100) to both partners when they knew they caused very severe harm to the partners. If the three pain levels were collapsed, the two-way interaction between the participant’s group and the partner’s compensation strategy was significant, *F*(1,72) = 5.93, *p* < 0.05, $\eta_{p}^{2}$ = 0.08. Pairwise comparison with Bonferroni correction showed that for the social group, the allocation was higher to the social partner than to the monetary partner, *F*(1,72) = 14.53, *p* < 0.001. However, for the monetary group the allocation to the two partners did not differ, *F* < 1, *p* > 0.1.

The main effect of partner’s compensation type was significant, *F*(1,72) = 8.74, *p* < 0.01, $\eta_{p}^{2}$ = 0.11. The participant compensated more for the social partner (3.76 ± 0.27) as compared with the monetary partner (3.32 ± 0.25). The main effect of pain-level was also significant, *F*(2,144) = 148.00, *p* < 0.001, $\eta_{p}^{2}$ = 0.67 (**Figure 5**). The participant compensated most for the high pain-level (5.75 ± 0.37), less for the low pain-level (3.61 ± 0.27) and least for the no pain level (1.26 ± 0.21). The main effect of participant group was also significant, *F*(1,72) = 8.15, *p* < 0.01, $\eta_{p}^{2}$ = 0.10, such that the social group compensated their partner (4.24 ± 0.35) more than the monetary group (2.83 ± 0.35).

## Discussion

Following interpersonal transgression, the transgressor may try to restore the relationship with the victim via certain forms of compensation. However, different forms of compensation are not equally effective for every individual in every social context. Here we showed that after being harmed, some people preferred to be compensated by money while others preferred non-monetary compensation, such as the transgressor sharing the harm. Moreover, the individual differences in preference for compensation not only had an impact on the victim’s perceived social distance toward the transgressors (participants felt closer to the transgressor whose compensation matched the their own preference) but also had an impact on the victim’s subsequent reciprocal behaviors toward the transgressors. Compared with previous investigations into guilt and compensation (e.g., de Hooge et al., 2007, 2011; de Hooge, 2012; Yu et al., 2014), the current study contributes two novel findings: first, we distinguish two types of compensation (communal versus exchange) that are commonly used in different social contexts as well as two subgroups of individuals who prefer different compensation strategies; second, we go one step further to show how individuals’ preference of certain way of compensation influences their own social relationship and reciprocal behaviors.

The progress made by the current study benefited from the interpersonal paradigm adopted here and in a few previous studies (e.g., Koban et al., 2013; Crockett et al., 2014, 2015; Yu et al., 2014). This paradigm has the strength of putting the participants in the social context and confronting them with the (ostensibly) real social consequences of their performance, choices, or decisions. The interpersonal paradigm allows us to investigate the psychological mechanisms of social emotions, interactions and relationships as they actually occur (rather than being limited to the participant’s imagination). Moreover, it is natural and convenient to include social modulations, such as communal versus exchange norms, in the interpersonal paradigm to broaden our understanding of the regularity underlying complex social interactions (Schilbach, 2014). As hypothesized, our results suggest that the participants’ preference of the manner of social interaction (e.g., communal versus exchange) did influence both their social relationships and reciprocal behaviors.

Monetary compensation is calculable and thus easy to precisely balance an inflicted harm. The downside is that money may readily trigger the monetary/exchange norm, which runs the risk of further dampening the social relationship (Heyman and Ariely, 2004; Ariely et al., 2008; Lacetera and Macis, 2010). The present study confirmed that money cannot repair all transgressions—or at least not better than interpersonal forms of compensation in certain cases. More importantly, we showed that individual differences in the victim’s preference for the manners of compensation influence the type of social relationships (communal versus exchange) that the victims formed with their former transgressors. Such individual differences were not only predictive of the types of relationship between the victim and the transgressor, but also influenced the victim’s perceived social distance and subsequent reciprocal behaviors toward the transgressor. Specifically, the victims who preferred “social” compensation (i.e., bearing pain) tended to form more communal (i.e., less exchange) relationships with and felt closer to the transgressors who compensated by bearing pain. In contrast, the victims who preferred “exchange” compensation (i.e., paying money) did not show any difference in social distance and prosocial behaviors toward different transgressors.

Several mechanisms may help us understand why the differences between the two compensation types occur mainly in the participants who preferred social/communal norms. One possibility is that the individual self-sufficiency orientation guides compensation behaviors. Self-sufficiency is defined as an insulated state wherein people put forth effort to attain personal goals and prefer to be separate from others. Money can make the self-sufficiency orientation more salient (Vohs et al., 2006). In this way, individuals who rely on monetary norms, compared with those who rely on social norms, feel less dependent on social connection and thus may care less about how others treat them. Another possibility is that exchange relationship orientation influences compensation behaviors. In light of the communal (or exchange) relationship theory, the individuals who have high exchange relationship orientation always keep precise balance in their social interactions (Mills and Clark, 1982). Equal and immediate return in the helping behavior draws clear boundary between the self and others, which decreases interpersonal risk but means less connections and less willingness to keep long-term relationships with others (Roberts, 2004). Thus, both self-sufficiency orientation and exchange relationship orientation reflect an individual’s need for independence from others, and a feeling of dependence may render an individual more sensitive to different types of interpersonal relationships or compensation strategies (Doi, 2014). In contrast, individuals who commit to exchange/monetary norms have a higher self-sufficiency orientation and exchange relationship orientation when compared with the social group, making them less likely to care about interpersonal information/intention conveyed through the partner’s compensation strategy.

As compensatory behaviors are mainly driven by emotions such as guilt (Baumeister et al., 1994; Ketelaar and Au, 2003; Yu et al., 2014), the guilty feeling and intention for restoring relationship inherent in the compensation behaviors (paying money or bearing noise) may affect individuals from the social group more than individuals from the monetary group. In our study, bearing harm for others may convey more empathy or care for partner’s pain compared with paying money. Thus bearing harm exhibited a more consolatory effect than money for individuals from the social group, making them compensate more points to their partners (McCullough et al., 2014). These results suggest that individuals who are inclined to adhere to the social/communal norm are more sensitive to different types of interpersonal relationships or compensation strategies than individuals adhering to the monetary/exchange norm.

Additionally, our studies showed that the social norm motivated the individuals from the social group through a strategy of matching. Specifically, when the offender’s compensation strategy (e.g., bearing harm) was consistent with the relationship type of the individuals from the social group (i.e., communal relationship), the individuals from the social group experienced more closeness with the offender and exhibited more compensatory behaviors in the subsequent role-reversed task than the offender who had compensated by paying money (i.e., the unmatched condition). What is the underlying mechanism for this matching process? A possible explanation appeals to the individuals’ self-verification motivation (Swann et al., 1989, 1994). This account proposes that people prefer to be seen in the same way as they see themselves. This motivation may incline individuals to prefer to be treated in the same way in which they treat others. Thus paying money to individuals from the social group may pose a conflict with those individuals’ self-verification motivations. This conflict may weaken the restoring effect of compensation. This explanation is line with the recent research on gift-giving that the matching between giver’s and the recipient’s characteristics can increase the effectiveness of gift-giving (Kube et al., 2012; Paolacci et al., 2015).

However, we did not find a significant incentive effect of money on individuals from the monetary group, which may have resulted from the small amount of money used in the current study. One-hundred points of tokens roughly amounted to $ 0.2, which may not be attractive enough to motivate these individuals. It has been shown that the intensity of the reinforcement plays a key role in determining the effectiveness of the reinforcers on social behavior (Gneezy and Rustichini, 2000; Heyman and Ariely, 2004). Future study should match the subjective values of the two types of compensation (paying money versus bearing noise). Another limitation of the current study is that we did not directly measure guilt in the second phase of the task. It is still an open and important question as to whether the communal/exchange relationship formed with different partners can influence one’s feelings of guilt toward the partners and whether such feeling can account for the difference in reciprocal/compensatory behavior.

In summary, we demonstrated that the effectiveness of compensation varies as a function of the individual differences in preferences for communal versus exchange modes of social interaction. The preference for a certain type of compensation (e.g., paying money versus bearing noise) also influences the interpersonal relationships formed between the recipient and the provider of the compensation: monetary compensation acts to undermine the perceived closeness with the recipient who weights communal norms more than exchange norms toward the provider of the compensation. These findings have implications for both institutional and interpersonal consolatory actions: for severe damage to social relationship, trust, cooperation and the like, material compensation alone may not be enough to heal the wounds, and may even make things worse (e.g., decreasing interpersonal closeness and reciprocal behaviors). In that case, a sincere apology or other authentic and creative social compensation strategies may be better ways of repairing the threatened interpersonal relationship.

## Conflict of Interest Statement

The authors declare that the research was conducted in the absence of any commercial or financial relationships that could be construed as a potential conflict of interest.

## Footnotes

1 The questions for social distance were adapted from Bogardus (1933). In the original work, the author asked the participants to evaluate the social distance expressed in 60 statements, ranging from “would marry” to “would have lived outside my country.” These were not meant to be a unified questionnaire and thus no reliability was reported. We selected two items from these statements that we believe were the most closely related to campus life.

2 The Cronbach’s α for the original questionnaire is 0.73 (Williamson and Clark, 1989).
